# Temporal patterns in the soundscape of the shallow waters of a Mediterranean marine protected area

**DOI:** 10.1038/srep34230

**Published:** 2016-09-28

**Authors:** Giuseppa Buscaino, Maria Ceraulo, Nadia Pieretti, Valentina Corrias, Almo Farina, Francesco Filiciotto, Vincenzo Maccarrone, Rosario Grammauta, Francesco Caruso, Alonge Giuseppe, Salvatore Mazzola

**Affiliations:** 1 National Research Council – Institute for Coastal Marine Environment – Bioacousticslab Capo Granitola, Via del Mare, 6 – 91021 Torretta Granitola, Campobello di Mazara (TP), Italy; 2 Department of Pure and Applied Sciences (DiSPeA) – University of Urbino– Campus Scientifico “Enrico Mattei”– 61029 Urbino, Italy; 3 ENEA – Observations and Analyses of Earth and Climate –Via Principe di Granatelli, 24 – 90139 Palermo, Italy

## Abstract

The study of marine soundscapes is an emerging field of research that contributes important information about biological compositions and environmental conditions. The seasonal and circadian soundscape trends of a marine protected area (MPA) in the Mediterranean Sea have been studied for one year using an autonomous acoustic recorder. Frequencies less than 1 kHz are dominated by noise generated by waves and are louder during the winter; conversely, higher frequencies (4–96 kHz) are dominated by snapping shrimp, which increase their acoustic activity at night during the summer. Fish choruses, below 2 kHz, characterize the soundscape at sunset during the summer. Because there are 13 vessel passages per hour on average, causing acoustic interference with fish choruses 46% of the time, this MPA cannot be considered to be protected from noise. On the basis of the high seasonal variability of the soundscape components, this study proposes a one-year acoustic monitoring protocol using the soundscape methodology approach and discusses the concept of MPA size.

Soundscape analysis is an emerging field of ecological research^1^ that contributes information about biological compositions and environmental conditions. In marine ecosystems, studies have underlined the importance of the acoustic environment to provide information about the quality and types of species habitats^2^,^3^,^4^,^5^.

The acoustic environment of a given habitat, or “soundscape”, includes the sounds produced by biotic, abiotic and anthropogenic activity^6^. These three components, defined as biophony, geophony and anthropophony, interact with each other and determine the peculiar and distinct underwater sound signatures^5^,^7^,^8^, which show a recognizable temporal pattern on daily and seasonal time scales^6^,^9^.

In marine shallow waters, biophonies are produced by fish, invertebrates and marine mammals. Marine animals emit sounds mainly for communication, and environmental recognition. In some cases, animals generate sounds involuntary during other activities (e.g., during swimming, grazing or shell movement). All these sounds contribute to the biophonic component of a particular soundscape^10^,^11^,^12^.

Vocal fishes produce impulsive or frequency-modulated sounds at low frequencies and low amplitudes, with differences in the duration and number of pulse trains for each species^13^. Invertebrates, such as shrimp, lobsters and bivalves, emit voluntary or involuntary impulsive and cracking broadband signals^10^,^14^,^15^. In coral reefs, snapping shrimp produce the dominant acoustic energy and exhibit clear daily acoustic trends^16^. These benthic-dwelling shrimp produce wideband pulses from 3 to 100 kHz, with an irregular pulse repetition rate^14^, which results from the rapid closing of their enlarged claws and the consequent collapsing cavitation bubble^17^. Marine mammals in Mediterranean coastal habitats, such as bottlenose dolphins, use two types of sound: broadband impulsive signals (clicks/burst), ranging from a few kHz up to 120 kHz^18^, and modulated narrowband whistles^19^,^20^. Biological sources included in a characteristic soundscape can be either transient^21^,^22^, show seasonal patterns^11^ or be resident^21^. Moreover, the occurrence of acoustic signals can be related to different ecological processes, such as reproduction, agonistic and territorial displays, detection of predators, searching and foraging for prey, orientation and navigation, and group cohesion^6^,^15^,^23^,^24^,^25^.

The abiotic sounds in coastal areas are determined by winds and waves, including breaking surface waves^11^, rainfall^26^, and waves beating against cliffs. The wind contribution dominates from a few Hz to 30 kHz, and surface waves cause mostly infrasonic noise at frequencies from 10 to 100 Hz^11^. The two sources are related, although surf noise, which is defined as wave noise localized near the land-sea surface, is prominent, even in calm wind conditions. Rainfall also produces energy peaks from 15–20 kHz^27^, while thunder and lighting generate sounds at lower frequencies, which contribute to background noise even if the storm is distant^28^.

Anthropogenic noise in coastal areas is mainly due to vessel traffic^29^, particularly at low frequencies (<1 kHz)^24^,^30^,^31^. Vessel traffic noise is primarily due to the cavitation and rotation of boat propellers^30^, as well as the operation of winches and other shipboard equipment^32^. As a consequence, boat disturbances change in relation to the type and size of vessel and its speed^5^,^7^,^8^,^31^,^33^. The increase in background noise over the past 50 years has been attributed to the growth of anthropogenic activities, particularly shipping traffic^30^,^32^. Many studies have demonstrated the effects of noise pollution on the communication, behaviour and physiological state of fish, marine mammals and crustaceans^34^,^35^,^36^. Shipping traffic noise can interfere with an animal’s ability to perceive a sound, which is defined as a masking effect. Masking can be complete, if the signal is not detected at all, or partial, if the signal is detectable by the listener but the content is difficult to understand^37^. Noise at the same frequency as that of biological sounds can produce different reactions in animal vocalizations, such as raising the intensity^38^ or changing the frequency of the vocalization^39^. Furthermore, in a receiver animal, masking might alter many vital functions, such as echolocation and the detection distance of a predator or conspecific^37^.

The Mediterranean Sea is affected by heavy traffic, and the Sicilian Channel is the principal path between the Eastern and Western basins. The Mediterranean Sea has high biodiversity, and its shallow waters, despite having a higher density of human activity (coastal artisanal fishery, recreational and tourist activities and ship traffic close to harbours), represent a crucial environment for the adults, juveniles and larvae of many marine organisms^40^. Although the formation of marine reserves helps to preserve some habitats (or a small portion of a habitat), high levels of noise pollution cannot be avoided. Indeed, acoustic energy can propagate beyond the boundaries of most no-entry zones of marine protected areas (MPAs), usually a few hundred metres. Mediterranean MPAs have been established to prevent their biodiversity from being deteriorated by human activities (above all fishing). Five key features contribute to the effectiveness of MPAs: no take, well enforced, old (>10 years), large (>100 km^2^), and isolated from deep water or sand^41^. Increasing the size of no-take zone increases the density of commercial fish within the reserve compared to out side^42^.

The MPA “Pelagie Islands” is located in the Strait of Sicily (see Fig. 1), which divides the Eastern and Western Mediterranean basins and divides Africa from Europe. The Sicilian Channel is considered an area of high biogeographical^43^ and hydrodynamic importance^44^, and it can be regarded as a privileged observatory for biodiversity monitoring^45^. The Pelagie Islands are an asset for the biodiversity of the Mediterranean. In their coastal waters, vulnerable (fin whale, bottlenose dolphin), endangered (common dolphin), and near-threatened species (brown meagre, *Sciaena umbra*) (IUCN Global Species Programme Red List Unit) live and/or migrate. Some authors^45^ have characterized the littoral fish assemblage of Lampedusa (the biggest island of the Pelagie Archipelago) to create a reference against which changes in the Mediterranean rocky-reef fish assemblage can be assessed in the future. In Lampedusa (Fig. 1), 23 families and 61 taxa of fishes have been recorded^45^, with a predominance of Labridae, including sonoriferous fishes, such as *Chromis chromis*, *Sciaena umbra*, and Gobidae. Galatheidae, Hyppolytidae and the three sonoriferous species of snapping shrimp belonging to the Alpheidae family (*Alpheus dentipes*, *Alpheus macrocheles*, and *Synalpheus gambarelloides*) have also been included in a checklist study^46^ on the decapod crustaceans living in Lampedusa from 0 to 30 m depth in rock and in the *Posidonia oceanica* substratum.

Characterization and evaluation of the contributions of natural and anthropogenic sources and identification of the ecological dynamics are crucial elements for assessing the impact of man-made disturbances on marine habitats^11^,^30^. The European Union Marine Strategy Framework Directive (MSFD)(2008/56/EC) promotes the achievement of a good quality environmental status for European waters by 2020. In particular, Descriptor 11.2 about “continuous low frequency sound” aims to monitor trends in the ambient noise level within the 1/3 octave bands^47^ of 63 and 125 Hz (centre frequencies) (re 1 μΡa rms). To obtain a baseline to develop a noise-monitoring plan in marine shallow waters (i.e., how long should the monitoring last? How many recording stations are needed to appropriately detect the spatial variability in the soundscape components? How do biological sound sources contribute to the noise level? How do the different soundscape components change in different seasons?), a one-year octave band analysis could provide a useful indication of the seasonal and circadian variability of the levels of noise and contribute to the detection of possible sources of noise, distinguishing them as anthropogenic or non-anthropogenic.

In recent years, automated tools and new ecoacoustic metrics have enabled the quantification of the amount of sound and the estimation of the level of biodiversity in terrestrial environments using large datasets^48^. For example, ecoacoustic metrics have been successfully used to study the alteration of singing dynamics caused by traffic noise^49^. These automatic procedures have the potential to provide useful insight when working with marine acoustic recordings, but until now, very few studies have applied these methodologies to the underwater world^5^.

This study explores the shallow water soundscape of an MPA in the central Mediterranean Sea (Lampedusa Island) over an entire year. The main aims were to a) investigate the seasonal and circadian patterns of octave band sound pressure levels (BPLs), b) identify the principal biological, physical and anthropogenic sound sources c) test the acoustic complexity index^48^ (ACI) as an automated metric to describe the biotic contribution to the soundscape, and d) quantify the percentage of time in which fish choruses are masked by vessel passage noise.

## Results

Table 1 summarizes the results of the BPL analysis for different seasons, distinguishing daytime (12:00 pm ± 2 hours), night-time (12:00 am ± 2 hours) or 24 hours. Figure 2 shows the monthly trends of the BPLs for the lower and higher frequencies. The BPLs at lower frequencies (Fig. 2a, from 63 Hz to 1000 Hz) increased from November to March. The higher frequencies (Fig. 2b, from 2 kHz to 64 kHz) followed the opposite pattern, with lower values during the winter. This difference in trends is appreciable in Fig. 3, in which the one-year mean power spectrum (black line) and the summer and winter mean power spectra (blue and grey lines, respectively) are shown. For the lower frequency (62 Hz), the difference between the winter and summer BPLs was 5.9 dB (respectively, 103.7 and 97.8 dB re 1 μPa, see Table 1), and for the higher frequency (i.e., 8 kHz) the mean difference between winter and summer was 8.6 dB (respectively, 102.3 and 110.9 dB re 1 μPa, see Table 1). This seasonal variability was mainly attributable to the sea state for the lower frequencies and to the activity of snapping shrimp for the higher frequencies (see Table 2, Figs 4 and 5). The total band sound pressure level is much more stable over seasons and with the circadian cycle (see Table 1). Figure 3 also shows a “stability” band centred at 2 kHz, which has a mostly stable and lower BPL throughout the year. This can be explained by the correlation values between BPLs at 2 kHz and the main components of the soundscape in Table 2. Excluding the anthropophony, a very low level of correlation of biophony and geophony vs. BPL at 2 kHz was found (Table 2).

In Fig. 4, the median power spectra for the night-time hours and daytime hours are shown for all seasons. Circadian patterns are more evident during the summer, both for the higher and the lower frequencies. During the other seasons, it is possible to distinguish some small differences (approximately 2 dB) between night and day only for the higher frequencies (from 4 kHz). In Fig. 5, the mean circadian trends for the BPL and ACI values for each month of the year are shown for three selected frequency bands (centred at 250 Hz, 1 kHz and 4 kHz). Figure 5a,b show the mean numbers of counted vocalizations per minute produced by fish (the grey area). The fish counting peaks at sunset for 250 Hz and 1 kHz are in line with the ACI peaks (blue line) and with the correlations in the octave bands occupied by fish sounds (as shown in Table 2). The BPLs (black line) showed the same peaks as the ACI, although there are other peaks during the day, probably due to vessel passages during July and August. The one-minute mean number of pulses produced by snapping shrimp showed a marked circadian pattern, with peaks during sunset and sunrise (Fig. 5c, grey area). The number of pulses and the circadian pattern decreased during the winter. Snapping shrimp sounds (Fig. 5c) were well-correlated with both the BPL and ACI values in the corresponding octave bands (Table 2; i.e., for 16 kHz: BPL and ACI vs. snapping r = 0.84 and 0.83, respectively).

Table 2 shows the correlation between the daily mean values of BPLs and the number of vessel passages from the 2 kHz octave band and above. In the same table, ACI does not show any correlation with vessel passages or wind, whereas BPLs are correlated below the 2 kHz octave bands.

Throughout the year, we recorded a mean of 13 vessel passages per hour. The analysis of the interference of vessel passage noise with the detectability of fish chorus showed that 46% of the files included vessel passages. Figure 6 shows the median (whisker box represents 40% of the data) BPLs for files with fish choruses and without vessel noise (green plot), with vessel passage noise (black plot) (46% of files), and with natural ambient sound, in which no fish choruses or vessel passage were audible/visible in the spectrogram. The median BPL of the fish chorus files (in the bands 62, 125, 250 and 500 Hz) was more than 5 dB above the background noise, whereas the BPL of files containing vessel passage overcame the BPL fish choruses in the octave bands below 2 kHz.

Sonar noise consisted of narrow band pulses at 50 or 30 kHz and was present in 3.3% of the files. Airgun pulses were recorded during April and March and were in 2.4% of the files for the entire year.

Figure 7 shows a general overview of the frequency band partition for the different components of the soundscape on two continuous recording days. Snapping shrimp (S) are represented by grey clouds up to 2.5 kHz and showed increased activity during sunset and sunrise. Fish choruses (F), which are below 1.5 kHz, are represented by the smallest grey clouds during sunset. Vessel passages (V), which very often masked all the frequency bands (see the vertical black lines), represented the strongest sound in the soundscape (blackest signals). The “silence band” between 1.5 and 2.5 kHz was interrupted by ship noise.

## Discussion

Seasonal trends are well defined for both the lower frequencies and higher BPLs (see Fig. 2). In the lower frequencies, sounds were much louder during the winter due to the physical noise of the waves caused by the increased wind speed^11^ (Table 2). The higher frequency BPLs (from 4 kHz) were dominated by snapping shrimp sounds that increased in the summer (Table 2, Figs 4 and 5c)^16^,^21^. As suggested by some authors^16^,^50^, this increase in snapping activity could be due to the temperature increase of the water (poikilotherm animals). The median power spectrum was different in the winter and summer, with an inversion of the levels between the higher and the lower frequencies (see Fig. 3). For the lower frequencies (below 2 kHz), the difference between the summer and winter was caused by the variation in wind speed between seasons. Because the lower frequencies and higher frequencies have opposite seasonal patterns, the total band sound pressure level varies less than the lower and higher BPLs over the seasons and with the circadian cycle (see Table 1).

In the power spectrum (Fig. 3), a “stability” point at a low sound level, centred at 2 kHz (octave band 1420–2840 Hz), was found throughout the year. This stability phenomenon is related to the fact that both wave noise and the sounds produced by snapping shrimp partially enter this band (see in the Table 2 the low values of r and the slope in the linear regressions of BPL vs. wind speed and BPL vs. snapping shrimp counts for 2 kHz). This acoustic niche is a portion of the frequency band that one or more species could occupy, so its voice is not masked^51^. In the future, one or more species could better exploit this acoustic space because this AMP is relatively young (12 years old). Alternatively, this silence band could have a special unknown function in the ecosystem, which should be investigated for other marine ecosystems. However, it is not possible to exclude that some propagation effect could create this lower level of noise centred at 2 kHz BPL.

Focusing on the circadian trends (see Figs 4 and 5), we noted a marked phenomenon for the higher frequencies due to the variation in the snapping shrimp noise, which was previously observed in other temperate seas^21^. Circadian trends in snapping sounds, with larger numbers and sound levels at night, are compatible with nocturnal increments in the activities of other marine crustaceans^52^. The circadian trend in frequencies above 4 kHz was greater during the summer, with peaks at sunset and sunrise. During the other seasons, the circadian pattern was still evident (Figs 4 and 5c). At lower frequencies, especially in the summer, there was a circadian pattern due to fish choruses, with a peak during sunset and nocturnal activity. The fish chorus period started in May and was more evident during July and August (see the peaks in the ACI values for the 250 and 500 Hz bands and the fish count in Fig. 5a,b and the linear regression parameters for the 125, 250 and 500 Hz bands in Table 2). The acoustic analysis of the fish choruses and their circadian pattern is in agreement with Picciulin *et al.*^53^ for the *Sciaena umbra* species. Sciaenids produce two types of calls: for reproduction during the spawning season in summer and for disturbances^54^. In our data, we noted reduced noise due to waves during the summer at lower frequencies, which represented an advantage for fish choruses, which were clearly audible over the background noise (see Fig. 4). During the winter, autumn and spring, the fish choruses (with a 62.5–1000 Hz BPL below 100 dB) should be often covered by geophonic noise, with a possible reduction of their acoustic effectiveness (see Fig. 4). However, during the summer, vessel passages reduced the effectiveness. During the peak of fish vocalization activity (between 07:30 pm and 11:30 pm), we found a masking effect of vessel passage noise on fish choruses 46% of the time (see Fig. 6).

The application of ACI to marine soundscape studies revealed its potential utility to discover biological pulsed signals, such as snapping and fish choruses, amidst continuous noise (vessel passage and wave noise). The ACI results were strongly correlated with the biotic elements in the relative frequency bands, and the ACI were not correlated with the geophonic or anthropophonic elements (see Table 2). Whereas standard analyses—automatic counts of fish vocalizations and snaps—could not be performed on recordings with noise and could need a prior phase of the selection of files, ACI can be applied to the entire dataset. ACI requires minor pre-processing effort and help in determining when biological sounds occurred all year, avoiding gaps in the data. The other non-impulsive biophonies with frequency modulation (which were present in the frequency band centred at 1 kHz), were not well detected, probably due to the frequency and temporal resolution used for the ACI calculations. The ACI parameters must be set correctly to perform the calculations, and two measurements may be needed to guarantee that all biophonic elements are extracted.

Although the recording site is a MPA, it cannot be considered to be protected from noise. In fact, this MPA presents heavy anthropogenic noise, with a mean of 13 vessel passages per hour over one year, and with a masking effect on the fish vocalizations below 2 kHz during July and August for approximately 46% of the time. The impact of this almost continuous noise (in addition to the less frequent impulsive noise from sonar, air guns and other sources) on different marine organisms could include biochemical and behavioural changes and could affect the fitness of many species over the long term^35^. However, most studies that have assessed the impact of noise on fish and crustaceans have been conducted in tanks, and *in situ* experimentation, although more complex, should be conducted. Passive acoustic monitoring allows studies on vocalization and changes due to noise^29^,^55^. In our data, we noted a masking effect by anthropogenic noise on fish choruses (see Fig. 6), which could affect the reproductive effectiveness of fish vocalization during the summer season. As confirmed by our data, anthropogenic sounds produce the highest amplitude and broadest bandwidth masking effects (see Fig. 7) in an ecosystem of partitioned bands, where animals, such as fish and snapping shrimp, tend to maintain their sound activity within narrow (or stable) niches to avoid overlapping with other species^51^.

The results of this soundscape study emphasize the need to revise the concept of marine protected area size (MPAs). Acoustic noise should be considered a pollution factor when drawing the boundaries of MPAs to preserve marine ecosystems. Considering that the acoustic energy in water propagates very well (low absorption coefficient and high speed), a small protected area of a few hundred metres is poorly protected from this type of pollution. In our data, where the minimum distance between the recorder site (no-entry zone) and the no-protection area was approximately 150 m, we found 13 vessel passages per hour. Furthermore, during the summer between 7:30 and 11:30 pm, at frequencies below 2 kHz, nearly half of the time (46%), noise pollution altered the natural soundscape, masking fish choruses (see Fig. 6). In addition, the MPA areas closer to the borders received a greater amount of noise than the inner part (where our recorder was positioned).

Lower frequency acoustic energy can travel for long distances compared to energy at higher frequencies^56^; therefore, noise produced by nearby vessels has higher frequency components than noise produced by more distant vessels. In this study, in some cases, the very wideband loud vessel noise, reaching recorder saturation (more than 165 dB re 1 μPa rms Sound Pressure Level), suggests that the vessels could be less than 150 m from the recorder. In those cases, passive acoustics could also provide a tool to monitor and quantify the compliance to the limits of remote marine protected areas.

These one-year monitoring results show the variability in BPL levels, which change with circadian and seasonal patterns. In the context of MSFD, the development of monitoring plans should consider the variability and the single contribution of different natural and anthropogenic sources present in the soundscape of a marine shallow water area. Finally, this study considers a small and homogenous area (this MPA has a length less than 2 km), but the use of multiple arrays of recorders must be used to address the spatial variation of the soundscape in bigger areas.

## Materials and Methods

### Study area

**Figure 1:**
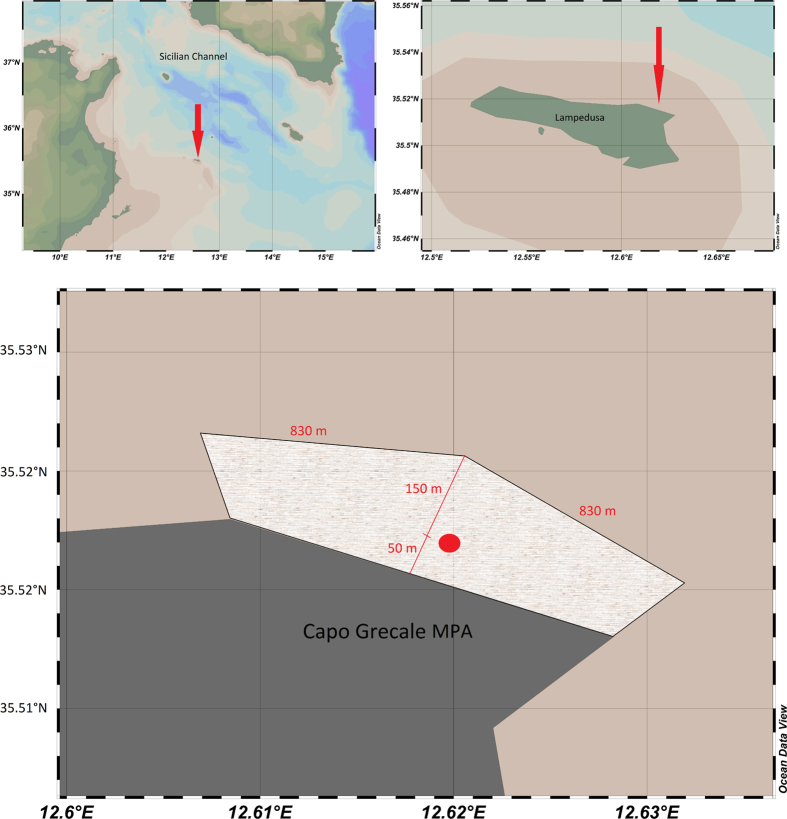
Top: The study area (red arrow) at two scales: Topleft-Lampedusa Island in the Central Mediterranean Sea; Topright-Lampedusa Marine Protected Area (red arrow). Below: Capo Grecale MPA delimited by yellow buoys with the spacing shown. The recorder position (red point) was near the midpoint of the MPA. (Map source: Schlitzer, R., Ocean Data View, odv.awi.de, 2015).

**Figure 2:**
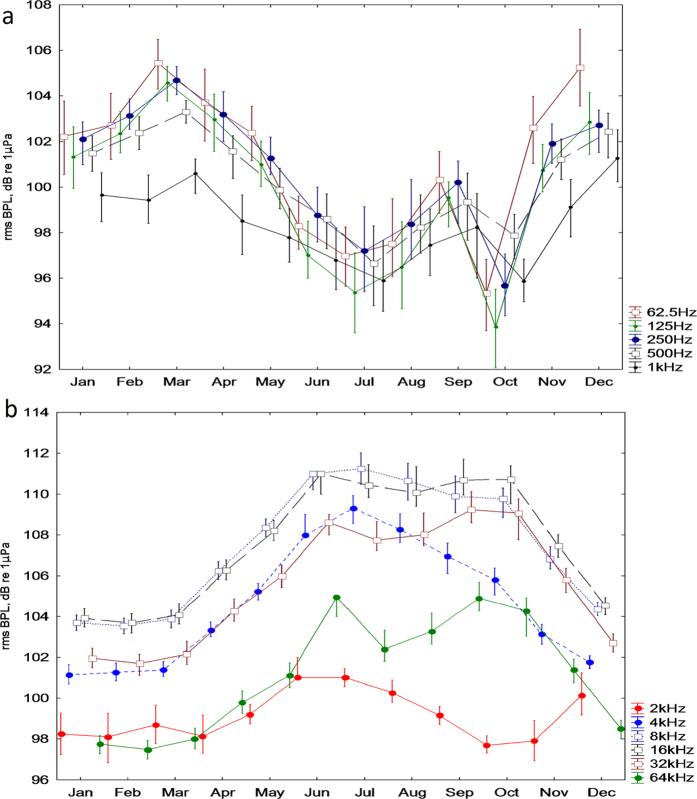
Seasonal trends (one year of data from July 2013 until June 2014) in the rms octave band sound pressure levels (BPL) for different frequencies. (**a**) Lower frequency BPL (from 62.5 to 1000 Hz). (**b**) Higher-frequency BPL from 2 kHz to 64 kHz. (Median; Whisker: 40^th^–60^th^).

Data were collected from July 1, 2013, to June 30, 2014, in the shallow waters of the Capo Grecale of Lampedusa, MPA Pelagie Island, Italy MPA. Lampedusa Island is located in the middle of the Mediterranean Sea and represents a point of unity and coexistence of flora and fauna of the warmer eastern basin and the west, which is influenced by Atlantic currents. In Capo Grecale, all human activities have been prohibited (no-entry zone) since 2002. The area covers a sea surface area of approximately 0.81 km^2^ (see Fig. 1). The bottom in this area is not uniform and consists of a mix of Mediterranean seagrass (*Posidonia oceanica*), sand and rocks.

### Data acquisition

The recorder was installed within the protected area with the permission of the MPA Committee. The data were collected using an autonomous recorder (SM2, Wildlife Acoustics, US) and hydrophone with a recording bandwidth of 8 Hz to 150 kHz and a sensitivity of −170 ± 5 dB re 1 V/μPa in the band of 25 Hz–100 kHz and −66 ± 1 dB re 1 V/μPa in the band of 100 Hz–15 kHz. The recorder site was selected to maximize the distance from the borders of the non-protected area and considering the limit of the coastline. The recorder was placed at the halfway point of the longer side (approximately 1600 m) of the Capo Grecale MPA (see Fig. 1), 50 m from a cliff and 150 m from the borders of the protected area, where all human activities are forbidden (Fig. 1; 35°31.27′N, 12°37.67′E). The minimum distance between the recorder and the allowed vessel transit area was approximately 150 m. The recorder was placed close to the seafloor (hydrophone height was 1.7 m from the bottom), at a depth of 25 m using a 35 kg ballast and a small sub-surface buoy to maintain the vertical arrangement in case of strong currents or bad weather. The buoy was connected to the upper part of the recorder with a thin rope (the distance between the buoy and hydrophone was 2 metres). All the components were connected with non-metallic rope to avoid noise due to moving parts. We set the sampling frequency to 192 kHz with a resolution of 16 bits, and no pre-amplification or filtering was applied during the recordings (except for an antialiasing filter). We sampled all day, setting a duty cycle of 2 minutes of recording (wav files) and 28 minutes of no recording. The recorder was recovered for maintenance every 3 months to change the batteries and storage memory.

### Data analysis

#### Circadian and seasonal octave band sound pressure levels (BPL) trends

For each 2-minute file, we calculated the average octave band sound pressure level (BPL, dB re 1 μPa, rms) beginning at the 62.5 Hz central frequency. In total, we calculated the BPL for 11 octaves: 63 (44–88), 125 (88–177), 250 (177–355), 500 (355–710), 1000 (710–1420), 2000 (1420–2840), 4000 (2840–5680), 8000 (5680–11360), 16000 (11360–22720), 32000 (22720–45440), and 64000 (45440–90880) Hz. For each file, the average total band sound pressure level was calculated (SPL, dB re 1 μPa, rms). We used a non-linear frequency band partition to obtain higher resolution at low frequencies, which showed greater variability than the high frequencies. Moreover, 1/3 octave band analyses were performed within the framework of Descriptor 11 of the European Marine Strategy Framework Directive 2008/56/EC11 for marine noise monitoring^47^. The automated BPL analyses were performed using SASLab software (Avisoft Bioacoustics, Glienicke, Germany).

We calculated the median BPLs for each month of the year. Considering the BPLs for each file, we calculated the median power spectra using all the files (one year of data), the files acquired during summer (from 20 June to 21 September), during winter (from 21 December to 19 March), during autumn (from 22 September to 20 December), and during spring (from 20 March to 19 June). The median power spectra were calculated for the daytime (12:00 pm ± 2 hours) and night-time (12 am ± 2 hours) for all seasons.

**Figure 3:**
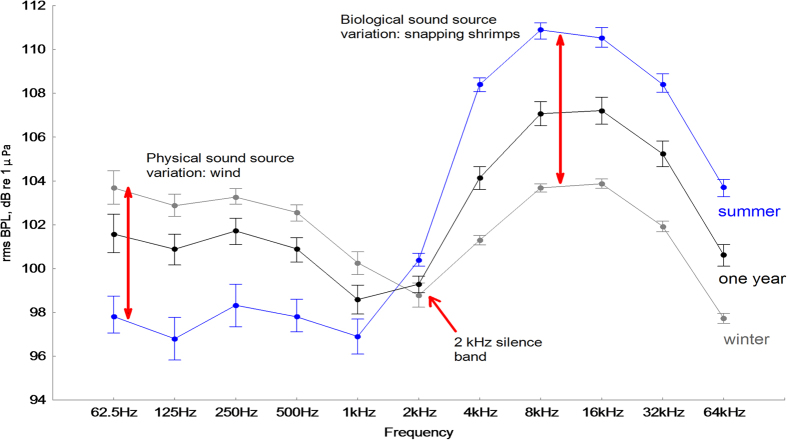
Seasonal trends of rms BPLs for all data (one year), summer data (July, August and September) and winter data (December, January and February)(Median; Whisker: 45^th^–55^th^ percentile). The differences between the summer and winter for each BPL are significant (Mann-Whitney U test, p < 0.001).

**Figure 4:**
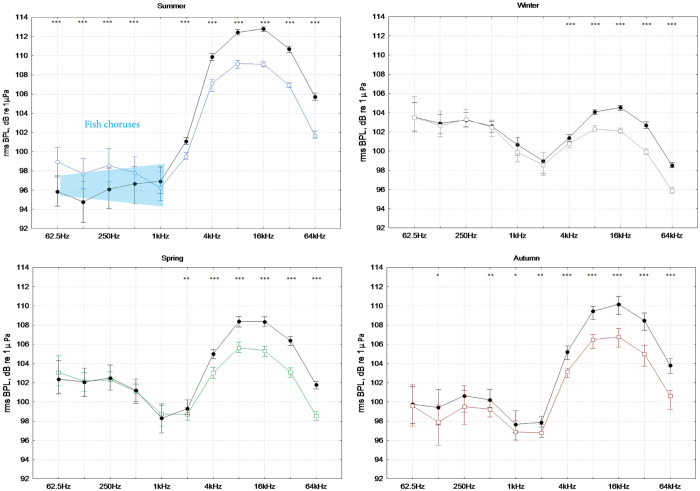
BPLs for the daytime (10 am to 3 pm)and night-time (10 pm to 03 am; black line) for all four seasons (Median; Whisker: 40^th^–60^th^ percentile). Differences between daytime and night-time (black lines) are marked with *for p-level < 0.05), **for p-level < 0.01, ***for p-level < 0.0001 (Mann-Whitney U test). The light blue polygon in the Summer graph represents the acoustic niche of fish.

**Figure 5:**
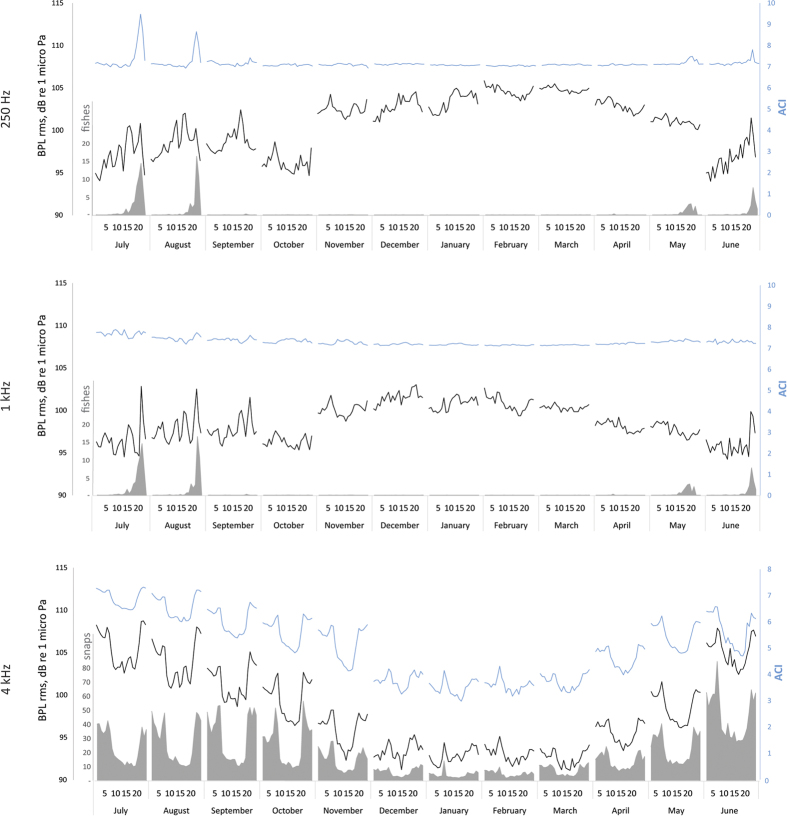
Circadian trends for each month in the BPLs (black line) and ACI (blue line) for three selected frequencies: 250, 1000 and 4000 Hz. For 250 Hz and 1 kHz, the fish vocalization counts per minute were added to the secondary y-axis (grey area). For 4 kHz, the snap counts per minute from snapping shrimp (grey area) were added to the secondary y-axis. X-axis: hour of the day for each month.

**Figure 6:**
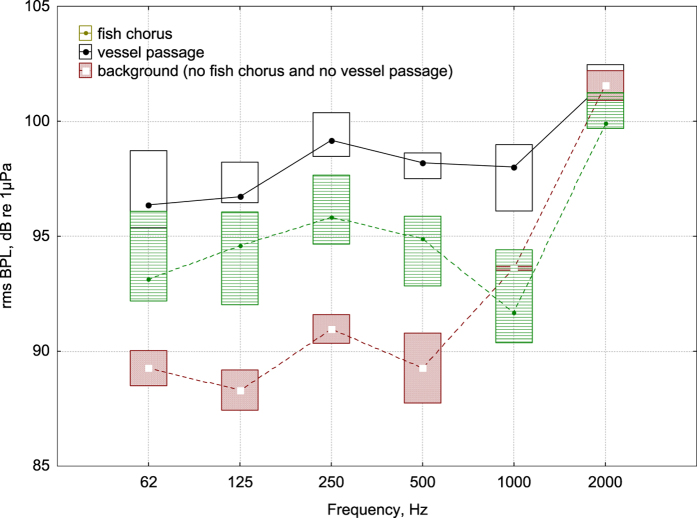
BPLs from 62 to 2000 Hz calculated for 10-day subsamples between 7:30 pm and 11:30 pm for July and August (the period in which we recorded the maximum fish vocalization activity). Median with 40% of the data (box) for fish choruses without vessel passage noise (green plot), with vessel passage noise (black plot) (46% of the recordings represented this condition), and for background noise (files with no fish chorus or vessel passage in the spectrogram) (median; Whisker: 30^th^–70^th^ percentile).

**Figure 7:**
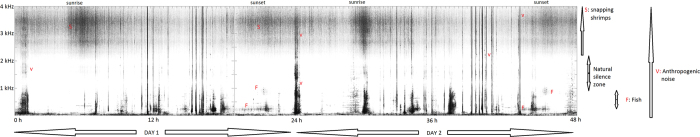
Two-day continuous spectrogram (11 and 12 July 2014) showing the snapping shrimp sound (S), fish choruses (F) and anthropogenic noise caused by the passage of vessels (V). The spectrogram was obtained using the XBAT software (Cornell University. USA). x-axis: time; y-axis: frequency 0–4 kHz; SPL intensity is shown in greyscale.

The Mann-Whitney U test (Statistica v.8 software package, USA) was used to assess significant differences between daytime and night-time during different seasons at different BPLs (Fig. 4) and between winter and summer (Fig. 3).

#### Complexity index analysis

To better characterize the biophonic component of the soundscape, we processed the dataset using the acoustic complexity index (ACI)^48^. The ACI is effective for terrestrial environments because it is minimally affected by constant sounds that have small amplitude variation over time (such as most of terrestrial anthropophony, e.g., cars and airplanes), while generating high values when computed for animal-produced sounds, which usually present high internal variability^48^,^57^. Like terrestrial environments, most underwater geophonies and anthropophonies (especially vessel traffic noise) are sounds that are composed of constant intensities over time (i.e., low complexity). Conversely, biological sounds are, in many cases, impulsive, such as snapping shrimp and fish vocalization (e.g., *Sciaena umbra*). Therefore, in this study, ACI was tested as a metric for detecting the possible presence of biological sounds, with the aim of isolating the biophony from the anthropophonic and geophonic components of the soundscape.

The ACI was computed using an automatic procedure to calculate the difference in amplitude (*I*) between adjacent temporal steps (*k*) using the following formula:


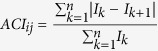


where *n* is the number of temporal steps (*k*), *i* is a frequency bin and *j* is the interval of time considered. The sum of all the frequency bins (*i*) and temporal intervals (*j*) was calculated for every recorded file.

To match the bands selected for the BPL analysis and to compare the results with the ACI output, we resampled the recordings at 181.760 Hz and successively applied a fast Fourier transform (FFT) of 8192 points. This enabled a resolution of 22.2 Hz (*i*) and 0.0454 seconds (*k*). For the 2 kHz octave band and above, we inserted an amplitude filter in the ACI calculations (SoundscapeMeter settings: noise filter = 5000 μV^2^/Hz) to avoid bias due to the sounds produced by snapping shrimp, which in certain periods of the year, were so dense that they were perceived by the ACI as one continuous sound, resulting in low complexity. Using the filter, the ACI was not applied to signals with amplitude lower than the selected threshold, and only the snapping events that were louder or closer to the hydrophone were considered. Because the filter for the ACI calculations was equally applied throughout the dataset, the quantity of louder/closer snaps was considered an indicator of the entire sound production of snapping shrimp. The filter was verified to correctly describe the snapping shrimp sound dynamics in a pilot study.

#### Main soundscape components: identification, counting, correlation, and interference

The files were analysed by an operator and/or by dedicated automated analyses to identify the main biological, physical and anthropogenic sound sources and to count each acoustic event. The circadian and seasonal patterns were correlated to the BPL and ACI values. The following procedures were followed for each main soundscape component:

- Biophonies-snapping shrimp: an automated analysis was used to count the number of snaps. After 2 kHz high-pass filtering, we performed “pulse train analysis” in the SASLab software package (Avisoft, Germany) to create the envelope of the acoustic wave and to count the pulses produced by the shrimp (settings for the envelope: rectification + exponential decay + decimation; settings for pulse detection: peak search with Hysteresis; other settings: Hysteresis = 20 dB; start and threshold = −10 dB; Threshold = 18; time constant = 1; resolution = 0.25 ms). For this analysis, subsamples of three days per month throughout the year were randomly chosen.
- Biophonies-teleost fish vocalization: an operator visually inspected (using the oscillogram and spectrogram) and listened to the files in the frequency band of 0–4 kHz to identify the impulsive fish vocalization. Pulse choruses were identified as *Sciaena umbra* vocalization because of the acoustic characteristics of the signals^53^,^54^ and the numerous sightings of specimens belonging to this species during the underwater maintenance operations of the recorders. We performed acoustic measurements of the fish pulses (first and second peak frequency in Hz, duration in s, 10^th^, 25^th^, 75^th^ and 90^th^ percentiles in Hz of the power spectral density distribution, 3 dB bandwidth in Hz) in a selection of files where the signals were not masked by noise from other sound sources (i.e., vessel passage, wave noise). An adapted version of a algorithm developed in the Matlab environment (Buscaino *et al.*^18^,^58^) was used to calculate these parameters. We applied k-medoids cluster analysis^59^, using the Mahalanobis metric, and two “median fish pulses” were selected. These signals were used as models for the successive analysis. A second Matlab script was developed to perform cross-correlation analysis (function xcorr^60^) to identify and count the fish pulses for one-year of data, comparing the two models of fish pulses with the unknown pulses extracted by the first Matlab code. An operator manually checked the results obtained from the automatic analysis, and a threshold of 0.9 for the cross-correlation value (from 0 to 1) was used to obtain less than 5% error in the identification of fish pulses.
- Geophonies-wave noise: the wind speed and direction were considered the principal factors that influence the sea state condition, and consequently, the noise created by breaking waves^11^ and waves beating against the cliff. For this analysis, a subsample of three days per month was randomly chosen. Only files with wind direction from the northern quadrant (0°–45° and 250°–360°) were considered for the analysis to avoid the shadow effect when the wind came from the land (in the southern quadrant).
- Anthropophonies-vessels, sonar, and air guns: an operator visualized (using the oscillogram and the spectrogram) and listened to selected files to count the number of vessel passages per file and the presence/absence of sonar and air gun pulses. The total number of vessel passages per day was then correlated with the daily mean BPLs. We considered days on which the average wind speed was less than 4 m/s to avoid noise due to bad sea conditions that could affect the accuracy of the analysis. We analysed a total of 89 days (4272 files), with a mean number of 7.5 days per month and a minimum of 4 days in March.

To define the correlation of the main soundscape components on the different BPLs and ACI, linear correlation analysis was performed using the Statistica v.8 software package (USA) (see Table 2).

To quantify the amount of time during which vessel passage noise interferes with the detectability of fish choruses, we considered the 62–2000 Hz BPLs (bands of fish vocalization) of files recorded between 7:30 pm and 11:30 pm in July and August (period in which we recorded the main fish vocalization activity). A subsample of 10 days in which the wind speed was less than 2 m/s was randomly chosen (total number of files = 90) to avoid differences in BPLs due to wave noise. We compared the BPLs of files with only fish choruses and the BPLs of files with vessel passages. As a baseline, we also considered the median BPLs for files without fish choruses or vessel passages.

Finally, two-day spectrograms were computed using the Matlab graphical interface code Xbat (Cornell Lab, USA) to provide a graphical view of the different soundscape components and the frequency bands they occupied.

## Additional Information

**How to cite this article**: Buscaino, G. *et al.* Temporal patterns in the soundscape of the shallow waters of a Mediterranean marine protected area. *Sci. Rep.* **6**, 34230; doi: 10.1038/srep34230 (2016).

## Figures and Tables

**Table 1:** rms octave band sound pressure level (Median, 10^th^percentile, 90^th^percentile) calculated for one year, for all seasons, for 24 hours, for the day (10:00 am−3:00 pm) and at night (10:00 pm−3:00 am).

	One Year									Summer									Autum									Winter									Spring								
	24 hours			day			night			24 hours			day			night			24 hours			day			night			24 hours			day			night			24 hours			day			night		
	m.	1 0 %	9 0 %	m.	1 0 %	9 0 %	m.	1 0 %	9 0 %	m.	1 0 %	9 0 %	m.	1 0 %	9 0 %	m.	1 0 %	9 0 %	m.	1 0 %	9 0 %	m.	1 0 %	9 0 %	m.	1 0 %	9 0 %	m.	1 0 %	9 0 %	m.	1 0 %	9 0 %	m.	1 0 %	9 0 %	m.	1 0 %	9 0 %	m.	1 0 %	9 0 %	m.	1 0 %	9 0 %
62.5 Hz	101.6	93.4	109.9	101.8	94.0	110.1	101.0	92.5	109.7	97.8	91.4	106.7	99.0	93.0	107.4	95.8	90.3	105.9	100.0	90.9	110.0	99.6	90.8	110.0	99.8	90.0	109.5	103.7	96.6	112.3	103.5	96.3	113.4	95.8	90.3	105.9	103.0	96.6	109.7	103.1	96.7	109.4	102.4	96.2	109.9
125 Hz	100.9	91.9	107.8	101.0	92.5	107.7	100.7	90.6	107.7	96.8	88.9	104.8	97.7	90.5	105.0	94.8	87.5	103.5	98.9	88.8	107.7	97.9	88.0	106.7	99.4	89.3	107.8	102.9	97.0	109.5	102.7	96.9	111.4	94.8	87.5	103.5	102.0	95.9	107.9	102.2	95.9	107.6	102.1	95.8	108.6
250 Hz	101.7	92.9	107.1	101.6	93.1	107.3	101.6	91.5	106.9	98.3	90.1	105.0	98.6	91.4	105.0	96.1	89.0	104.0	100.2	91.2	106.8	99.5	90.8	105.9	100.7	90.2	107.0	103.3	98.0	108.6	103.3	97.2	112.5	96.1	89.0	104.0	102.3	95.9	107.3	102.3	96.2	106.9	102.5	95.9	107.7
500 Hz	100.9	93.3	105.8	100.8	93.8	105.7	100.9	92.3	105.5	97.8	89.9	104.4	97.8	91.1	104.4	96.7	88.8	103.8	99.9	93.8	106.2	99.2	93.7	105.6	100.2	92.8	106.4	102.6	98.3	107.1	102.5	97.5	110.5	96.7	88.8	103.8	101.0	95.2	105.1	101.1	95.1	104.9	101.2	95.4	105.2
1 kHz	98.6	92.7	104.4	98.4	92.6	104.2	98.6	92.6	104.2	96.9	91.0	104.0	96.2	91.0	103.2	96.9	91.4	103.5	97.5	93.2	104.4	96.9	93.0	103.9	97.7	92.4	104.6	100.3	95.3	105.9	99.9	94.7	109.1	96.9	91.4	103.5	98.3	92.5	103.3	98.8	92.4	102.6	98.3	92.9	103.6
2 kHz	99.3	95.4	103.0	98.8	95.2	103.2	99.7	95.5	102.8	100.4	97.9	103.0	99.5	97.1	103.5	101.1	99.0	102.9	97 8	95.0	101.9	96.8	94.2	101.6	97.9	95.2	101.7	98.8	94.4	104.2	98.6	94.6	105.7	101.1	99.0	102.9	99.0	96.0	102.5	98.7	95.9	101.8	99.3	96.1	102.8
4 kHz	104.1	100.3	109.7	103.3	99.7	108.0	105.0	100.7	110.3	108.4	105.4	111.5	107.1	104.4	110.8	109.9	107.5	111.8	104.1	101.2	107.4	103.2	99.8	104.8	105.2	101.9	107.3	101.3	99.3	104.1	100.8	98.5	104.8	109.9	107.5	111.8	104.2	101.4	107.4	103.0	100.5	106.4	105.0	102.4	107.6
8 kHz	107.1	102.8	112.0	105.7	101.8	109.9	108.5	103.6	112.8	110.9	107.8	113.4	109.2	106.9	111.5	112.4	110.8	114.0	107.7	104.5	111.2	106.5	103.0	108.1	109.4	106.0	111.2	103.7	101.6	105.7	102.3	100.8	104.5	112.4	110.8	114.0	107.2	104.2	110.4	105.6	103.0	108.7	108.4	105.7	110.6
16 kHz	107.2	102.8	112.3	105.4	101.6	109.6	108.5	104.0	113.0	110.5	108.0	113.6	109.1	107.0	110.4	112.8	111.2	113.9	108.3	104.7	112.2	106.8	103.2	108.8	110.2	106.7	112.4	103.9	101.5	105.9	102.1	100.4	103.9	112.8	111.2	113.9	107.2	103.9	110.3	105.4	102.7	108.2	108.4	105.9	110.5
32 kHz	105.2	100.8	110.3	103.2	99.3	107.4	106.7	102.2	111.1	108.4	105.8	111.6	106.9	104.9	108.6	110.7	108.9	112.2	106.6	102.6	110.5	105.0	101.2	107.1	108.5	105.3	110.8	101.9	99.3	104.1	99.9	98.3	101.7	110.7	108.9	112.2	105.1	101.9	108.1	103.1	100.5	106.0	106.4	104.1	108.4
64 kHz	100.6	96.6	105.5	98.7	95.4	102.8	102.1	98.0	106.4	103.7	100.7	107.0	101.7	100.1	104.2	105.7	103.8	107.6	102.0	98.2	105.7	100.6	96.9	102.3	103.8	101.0	106.1	97.7	95.4	99.9	95.9	94.7	97.4	105.7	103.8	107.6	100.5	97.6	103.6	98.6	96.4	101.0	101.8	99.6	103.6
Totalband	116.3	112.7	121.2	115.6	112.1	121.2	117.3	112.9	121.4	117.9	115.6	120.1	116.1	115.3	119.5	118.7	117.8	120.2	116.5	114.2	120.6	115.0	113.7	120.2	117.0	115.0	120.5	114.5	111.5	122.4	114.0	110.9	123.2	118.7	117.8	120.2	115.8	113.2	122.2	115.0	112.5	121.9	116.3	113.7	122.7

**Table 2:** Linear regression equations, r (measure of the goodness-of-fit of the linear regression) and p-values (significance test for linear regression) for BPL and ACI versus biophonies (pulses caused by snapping shrimp and fish vocalization), geophonies (wind speed as the principal factor affecting wave height), and anthropophonies (vessel passage).

BPL/ACI octave band, Hz	Biophonies				Geophonies		Anthropophonies	
	BPL vs. Log10 Snapping shrimp counting	ACI vs. Snapping shrimp counting	BPL vs. log10 (fish sound counting)	ACI vs. fish sound counting	BPL vs. Wind speed^1^, m/s	ACI vs. Wind speed^1^, m/s	BPL vs. log10 vessel passages	ACI vs. log10 vessel passages
62.5	BPL = 102−1.3*S	ACI = 7 + 0.0*S	BPL = 99−2.1*F	ACI = 7 + 0.001*F	BPL = 95 + 0.9*w	ACI = 7 + 0.03*w	BPL = 93 + 2.3*V	ACI = 7.2−0.3*V
	r = −0.09	r = 0.00	r = −0.38	r = 0.10	**r = 0.58**	r = 0.21	r = 0.23	r = −0.40
	p = **	p = 0.94	p = ***	p = 0.09	p = ***	p = *	p = 0.30	p = **
125	BPL = 102−1.9*S	ACI = 7 + 0.2*S	BPL = 97−1.8*F	ACI = 7 + 0.01*F	BPL = 94 + 0.8*w	ACI = 7 + 0.00*w	BPL = 92 + 1.9*V	ACI = 7.5−0.4*V
	r = −0.13	r = 0.17	r = −0.26	**r = 0.68**	**r = 0.54**	r = 0.02	r = 0.13	r = −0.40
	p = ***	p = ***	p = **	p = ***	p = ***	p = 0.79	p = 0.57	p = 0.08
250	BPL = 102−1.2*S	ACI = 7 + 0.3*S	BPL = 98−1.0*F	ACI = 7 + 0.01*F	BPL = 96 + 0.7*w	ACI = 7–0.04*w	BPL = 97−0.5*V	ACI = 7.3−0.1* V
	r = −0.09	r = 0.21	r = −0.14	**r = 0.85**	**r = 0.50**	r = −0.17	r = −0.04	r = −0.06
	p = **	p = ***	p = *	p = ***	p = ***	p = *	p = 0.87	p = 0.60
500	BPL = 102−1.16*S	ACI = 7 + 0.2*S	BPL = 97−1.7*F	ACI = 7 + 0.01*F	BPL = 96 + 0.6*w	ACI = 7−0.03*w	BPL = 96−0.9*V	ACI = 7.3−0.1* V
	r = −0.10	r = 0.19	r = −0.24	**r = 0.72**	**r = 0.53**	r = −0.20	r = −0.06	r = −0.07
	p = **	p = ***	p = *	p = ***	p = ***	p = *	p = 0.79	p = *
1000	BPL = 98 + 0.1*S	ACI = 7 + 0.2*S	BPL = 96 + 0.1*F	ACI = 8 + 0.001*F	BPL = 95 + 0.7*w	ACI = 8−0.031*w	BPL = 96−0.2*V	ACI = 7.3−0.2* V
	r = 0.01	r = 0.25	r = 0.03	r = 0.12	**r = 0.56**	r = −0.37	r = −0.02	r = 0.10
	p = 0.57	p = ***	p = 0.66	p = *	p = ***	p = ***	p = 0.92	p = 0.37
2000	BPL = 96 + 2.3*S	ACI = 3 + 0.7*S	BPL = 101−0.2*F	ACI = 4 + 0.001*F	BPL = 99 + 0.1*w	ACI = 4 + 0.076w	BPL = 98 + 2.6*V	ACI = 2.3 + 0.6* V
	r = 0.32	r = 0.27	r = −0.07	r = 0.05	r = 0.16	r = 0.28	**r = 0.57**	r = 0.16
	p = ***	p = ***	p = 0.22	p = 0.36	p = *	p = ***	p = *	p = 0.15
4000	BPL = 97 + 5.6*S	ACI = 2 + 0.2*S	BPL = 109 + 0.5*F	ACI = 7 + 0.002*F	BPL = 108−0.2*w	ACI = 6−0.047*w	BPL = 107 + 1.8*V	ACI = 5.8−0.1* V
	**r = 0.72**	**r = 0.70**	r = 0.22	r = 0.33	r = −0.19	r = −0.17	**r = 0.51**	r = −0.01
	p = ***	p = ***	p = *	p = ***	p = *	p = *	p = *	p = 0.95
8000	BPL = 99 + 6.3*S	ACI = 1 + 2.7*S	BPL = 111 + 0.7*F	ACI = 7 + 0.002*F	BPL = 110−0.1*w	ACI = 6−0.008*w	BPL = 110 + 1.3*V	ACI = 6.5−0.6* V
	**r = 0.83**	**r = 0.78**	r = 0.36	R = 0.31	r = −0.10	r = −0.03	**r = 0.56**	r = 0.10
	p = ***	p = ***	p = ***	P = ***	p = 0.11	p = 0.64	p = *	p = 0.39
16000	BPL = 99 + 6.6*S	ACI = −0.2 + 2.9*S	BPL = 110 + 0.9*F	ACI = 5 + 0.004*X	BPL = 110 + 0.0*w	ACI = 5 + 0.004*w	BPL = 110 + 1.1*V	ACI = 5.5−0.8* V
	**r = 0.84**	**r = 0.77**	r = 0.45	R = 0.36	r = 0.02	r = 0.01	**r = 0.59**	r = −0.12
	p = ***	p = ***	p = ***	P = ***	p = 0.74	p = 0.85	p = *	p = 0.29
32000	BPL = 97 + 6.6*S	ACI = −1.1 + 2.1*S	BPL = 108 + 1.0*F	ACI = 3 + 0.005*F	BPL = 108 + 0.1*W	ACI = 2 + 0.024*w	BPL = 107 + 1.1*V	ACI = 3.1−0.7* V
	**r = 0.83**	**r = 0.73**	r = 0.45	r = 0.22	r = 0.08	r = 0.07	**r = 0.53**	r = −0.13
	p = ***	p = ***	p = ***	p = ***	p = l0.22	p = 0.27	p = *	p = 0.27
64000	BPL = 93 + 6.2*S	ACI = −0.35 + 0.4*S	BPL = 102 + 1.0*F	ACI = 0 + 0.001*F	BPL = 103 + 0.1*w	ACI = 0 + 0.019*w	BPL = 102 + 1.0*V	ACI = 0.6−0.2* V
	**r = 0.82**	**r = 0.64**	r = 0.45	r = 0.33	r = 0.15	r = 0.19	**r = 0.51**	r = −0.14
	p = ***	p = ***	p = ***	p = ***	p = *	p = *	p = *	p = 0.21

The data are from a subsampling of three days per month unless otherwise specified.

1Calculated only if wind direction was from the northern quadrants (0°–45° and 250°–360°).

p < 0.05→*; p < 0.001→**; p < 0.000→***.

Characters in bold indicate p-value < 0.05, r > 0.49.
